# One thousand simple rules

**DOI:** 10.1371/journal.pcbi.1006670

**Published:** 2018-12-20

**Authors:** Philip E. Bourne, Fran Lewitter, Scott Markel, Jason A. Papin

**Affiliations:** 1 Data Science Institute, University of Virginia, Charlottesville, VA, United States of America; 2 Bioinformatics and Research Computing, Whitehead Institute, Cambridge, MA, United States of America; 3 Dassault Systèmes BIOVIA, San Diego, CA, United States of America; 4 Department of Biomedical Engineering, University of Virginia, Charlottesville, VA, United States of America

What began as a one-off in 2005 as Ten Simple Rules for Getting Published [1] has, in thirteen years, now multiplied a hundredfold to become One Thousand Simple Rules for many aspects of one’s professional development and led to Quick Tips in the journal’s Education section. This milestone of a thousand rules has been reached thanks to the unselfish work of all stakeholders—authors, editors, reviewers, and readers. Let’s face it, writing, editing or reviewing a Ten Simple Rules (TSR) article is not the same as a publication that advances a scientific field. What it is going to get you is the satisfaction of knowing you have passed on a part of your experience in a form that is easily understood and acted upon by those following in your footsteps and hence have a very different kind of positive impact on science. Thank you. On the other hand, as a reader the TSRs might contribute in some small way to you getting tenure, or whatever else you care about in your professional life.

*Insight for impactful publications*. *Thank you @PLOSCompBiol, this is great for my students as they start writing*!

@jkellogg916

These are our words. In the summer of 2018 PLOS sent out a survey to both the authors and readers of TSRs to solicit your thoughts on the series. A summary of some of your words is presented in Table 1 and Table 2.

**Table 1:** What readers are saying about TSR that influenced them in their career.

*“It is difficult to choose one particular article as a favorite*. *I would like to point out that*, *as I progressed from being a Master's student doing research to a more experienced graduate student nearing graduation*, *a different set of 10SR articles have guided/influenced me*.*”* Sarvesh Nikumbh, Graduate student
*“How to write a review*, *by M Pautasso*. *It changed my approach to writing reviews*.*”* Anonymous, Academic faculty
*“Ten Simple Rules for Reproducible Computational Research*, *and other articles related to computational biology*, *programming*, *etc*. *They really helped in organizing my projects and code more efficiently*.*”* Endre Sebestyén, Postdoc

**Table 2:** What authors are saying about writing a TSR article.

*“It led to the collaboration with a sociologist (J*. *Evans)*, *and was highly discussed on social media*, *increasing the visibility of my laboratory*.*”* Stefano Allesina
*“I want to continue developing ways to support undergraduate research because I feel that researchers don't effectively use or train undergrads and take them for granted*.*”* Ben Harris
*“We simply wanted to share our experience in setting up a postdoc association*, *and discuss postdoc life in general*. *Also*, *this seemed like a good opportunity to have an "official track record" in leadership skills as a postdoc*.*”* Endre Sebestyén
*“After having read other TSR that I found really useful*. *I wanted to take the challenge of synthesizing my ideas on the topic I chose (better figures)*.*”* Nicolas Rougier
*“Writing our article (Ten Simple Rules for biologists learning to program) helped me build confidence in myself as a mentor and teacher*.*”* Maureen Carey

The story of how the TSRs started was outlined in that first article [1]. Suffice it to say here that the articles proved then, as now, a succinct and easily digestible piece of advice. Qualitative measures of success of the TSRs appear as framed versions on the walls in labs, or in the case of one of us (PEB) a stand in the corridor outside the lab which contained the collection of TSRs which periodically needed to be replenished. The fact that the stand was located near the bathroom and was eventually stolen will be left unexplored. The definitive qualitative measure of success came with the “Ten Simple Rules for Writing Ten Simple Rules” [2].

**Fig 1:**
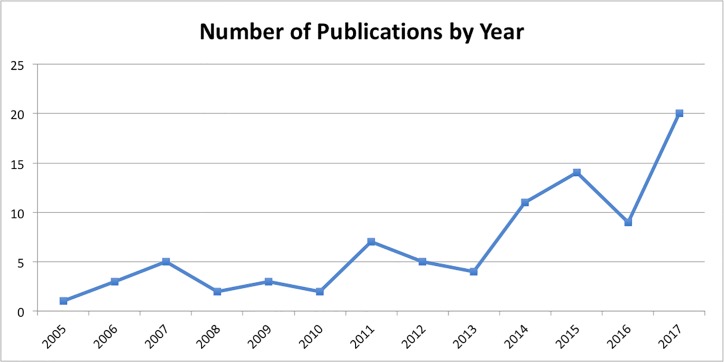
Number of TSR articles published per year.

A more quantitative measure of success comes from the growth of the series (Fig 1) and the article level metrics (ALMs) that PLOS keeps on each article. As of October 2018, there have been a combined total of 8.3 million views and downloads of the TSRs from the PLOS and PubMedCentral (PMC) Websites. Twelve articles each have over 100,000 views and downloads with “Ten Simple Rules for Writing a Literature Review” [3] topping the list with a staggering 1.1 million combined views and downloads. While not consistently translated, various articles have been made available in at least Spanish, Japanese, Russian, Norwegian, Chinese, and Farsi.

*I'm addicted to Ten simple rules in @PLOSCompBiol* ! *#reading how to making research software bulletproof*

@TivadarDanka

**Fig 2:**
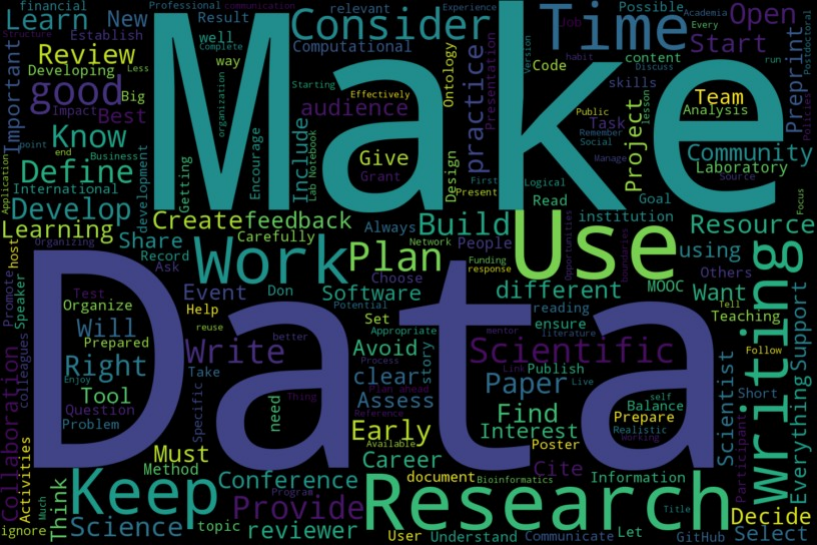
Word cloud derived from the text of TSRs. Courtesy of Andrew Lonsdale, November 2018.

It’s hard to capture the breadth of what has been covered in these one thousand rules (Fig 2). Perhaps the best way is with Ten Rules, well actually ten ways to subclass the one hundred articles. Those divisions are:

1. Self study/learning habits
2. Scientific communication
3. Career development and choices
4. Event planning
5. Education and mentoring
6. Using technology
7. Programming and software management
8. Service
9. Collaboration from local to international
10. Practicing more disciplined and organized science

With this level of coverage, has the series run its course? Is there nothing new to say? A question we have asked at PLOS a number of times only to have it answered by a new and novel submission. Certainly with one thousand rules novelty becomes an issue. Then again, in just reading responses to the reader survey which asked for novel TSR suggestions new ideas emerged—unique features of working for an NGO such as a non-profit foundation; reducing your carbon footprint while doing research; lab management; ensuring a work-life balance—and so on. Moreover, technologies, protocols, expectations, how we conduct science—think social media—are changing rapidly which calls for topics to be refreshed. In short, there would seem to be no end in sight.

There are also the questions of scope and form of dissemination. Many of the rules are not confined to computational biology and are clearly read by those outside our field. Since 2005 social media has become mainstream and while articles get tweeted are they more appropriate as blog postings? For now, we (PLOS and the TSR Editors), based on comments received through the surveys, feel we should keep the series going as is, but as a community journal your ongoing input is critical. What do you think comes next?
